# Detoxification Response of *Pseudomonas fluorescens* MFAF76a to Gaseous Pollutants NO_2_ and NO

**DOI:** 10.3390/microorganisms10081576

**Published:** 2022-08-05

**Authors:** Thibault Chautrand, Ségolène Depayras, Djouhar Souak, Mathilde Bouteiller, Tatiana Kondakova, Magalie Barreau, Mohamed Amine Ben Mlouka, Julie Hardouin, Yoan Konto-Ghiorghi, Sylvie Chevalier, Annabelle Merieau, Nicole Orange, Cécile Duclairoir-Poc

**Affiliations:** 1 Research Unit Bacterial Communication and Anti-Infectious Strategies (UR CBSA), University of Rouen Normandy, 55 Rue Saint-Germain, 27000 Evreux, France; 2 Praxens, Normandy Health Security Center, 55 Rue Saint-Germain, 27000 Evreux, France; 3 LPS-BIOSCIENCES SAS, Domaine de l’Université Paris Sud, Bâtiment 430, Université Paris Saclay, 91400 Orsay, France; 4 Polymers, Biopolymers, Surface Laboratory, University of Rouen Normandy, INSA, CNRS, Bâtiment DULONG—Bd Maurice de Broglie, CEDEX, F-76821 Mont-Saint-Aignan, France; 5 PISSARO Proteomic Facility, IRIB, F-76820 Mont-Saint-Aignan, France

**Keywords:** NO_2_, *P. fluorescens*, cell morphology, membrane, membrane integrity

## Abstract

Bacteria are often exposed to nitrosative stress from their environment, from atmospheric pollution or from the defense mechanisms of other organisms. Reactive nitrogen species (RNS), which mediate nitrosative stress, are notably involved in the mammalian immune response through the production of nitric oxide (NO) by the inducible NO synthase iNOS. RNS are highly reactive and can alter various biomolecules such as lipids, proteins and DNA, making them toxic for biological organisms. Resistance to RNS is therefore important for the survival of bacteria in various environments, and notably to successfully infect their host. The fuel combustion processes used in industries and transports are responsible for the emission of important quantities of two major RNS, NO and the more toxic nitrogen dioxide (NO_2_). Human exposure to NO_2_ is notably linked to increases in lung infections. While the response of bacteria to NO in liquid medium is well-studied, few data are available on their exposure to gaseous NO and NO_2_. This study showed that NO_2_ is much more toxic than NO at similar concentrations for the airborne bacterial strain *Pseudomonas fluorescens* MFAF76a. The response to NO_2_ involves a wide array of effectors, while the response to NO seemingly focuses on the Hmp flavohemoprotein. Results showed that NO_2_ induces the production of other RNS, unlike NO, which could explain the differences between the effects of these two molecules.

## 1. Introduction

Air pollution is one of the leading causes of mortality in humans, responsible for about 7 million deaths per year around the world [1]. This pollution is mainly caused by anthropogenic activities, which release pollutants in the atmosphere through the combustion of fuel in transports and industries [2] and the spreading of nitrogen fertilizers [3]. Among the pollutants produced by fuel combustion are reactive nitrogen species (RNS), especially nitric oxide and nitrogen dioxide. RNS are highly reactive molecules such as nitric oxide (NO^•^), nitrogen dioxide (NO_2_) and peroxynitrite (ONOO^−^), which can react with a wide range of biomolecules. NO^•^ can damage DNA through nitrosative deamination of bases and oxidative modifications of deoxyribose sugar [4], NO_2_ can induce lipid peroxidation [5] and ONOO^−^ can lead to various protein alterations such as tyrosine nitration [6]. Therefore, RNS can threaten the cellular homeostasis of biological organisms at high concentrations. The excess of RNS in the cell leads to a process called nitrosative stress. In humans, NO_2_ can induce reversible effects on health after 1 h of exposure to 5 ppm, and irreversible effects after 1 h at 45 ppm; the World Health Organization annual guideline for this molecule is 0.1 ppm [7,8].

RNS are therefore encountered by bacteria in polluted environments, but also during the immune response through the nitric oxide synthase pathway (NOS), where macrophages generate a burst of ROS and RNS to eliminate pathogens [9,10]. Resistance to RNS is therefore often an important part of the infectious process [11,12]. Furthermore, NO possess a signaling role in the formation of biofilm by various bacterial genera including *Pseudomonas* [13], where it acts as a biofilm disperser. The biofilm dispersing activity of NO has notably been used as an adjunctive therapy in the treatment of *P. aeruginosa* infections in Cystic Fibrosis cases [14]. Gaseous distribution inside biofilms and its consequences are complex as gas repartition varies between the liquid layer and the gaseous layer of biofilms exposed to air through mass transfer [15,16].

As such, it is important to understand the effects of environmental RNS on airborne bacteria and their defense mechanisms to better assess this indirect impact of air pollution on human health. One such mechanism is achieved by the Hmp flavohemoprotein, which possesses the ability to convert NO into nitrate aerobically [17], as well as a alkyl hydroperoxide reductase activity giving it a protective role towards membranes [18]. In *Pseudomonas aeruginosa*, the Hmp homolog Fhp is under the control of the NO-inducible activator FhpR [19].

*Pseudomonas fluorescens* is an extremely versatile bacteria able to grow in a multitude of environments including water, soil and air [20] The strain of *P. fluorescens* MFAF76a is an airborne strain possessing virulence factors such as the ability to form biofilms at 37 °C, the secretion of lipases and proteases and a high lytic activity on the human pulmonary cell line A549 [21]. These factors make MFAF76a a relevant model to assess the response of airborne bacteria to RNS. Previous work [22] has shown that 45 ppm of NO_2_ induces various modifications of the bacterial envelope and divisome. The objectives of this study are to assess the differences between the impacts of NO_2_ and NO, and the potential derivative species they generate on the physiology of MFAF76a and to analyze the bacterial detoxification response to these pollutants.

## 2. Materials and Methods

### 2.1. Bacterial Strain, Plasmids and Culture Conditions

Bacterial strains and plasmids used in this study are listed in Table 1.

Strains were grown overnight in Luria–Bertani (LB; DifcoTM BD 244620, Fisher scientific, Illkirch, France) medium broth under limited agitation (180 rpm). *P. fluorescens* strains were grown at 28 °C and *Escherichia coli* strains at 37 °C. Media were supplemented with antibiotics, as appropriate: 15 µg/mL (*E. coli*) or 50 µg/mL (*P. fluorescens*) gentamycin (G1264, Sigma-Aldrich, Saint-Quentin-Fallavier, France), 50 µg/mL (*E. coli*) or 100 µg/mL (*P. fluorescens*) kanamycin (10106801001, Sigma-Aldrich). The cultures of strains carrying pPSV35 plasmid were supplemented with 100 µg/mL IPTG (I6758, Sigma-Aldrich) for gene expression. For gaseous exposure, *Pseudomonas* cultures were diluted (OD_580_ nm = 0.08) in Davis Medium Broth (DMB), a minimal medium with 2.16 g/L glucose (Merck™ 1083371000, Darmstadt, Germany,) as carbon source [27]. The incubation was performed at 28 °C under agitation to reach the stationary growth phase. Bacterial cultures were transferred on cellulose nitrate membrane filters (0.45 μm, pore size 0.2 μm, diameter 47 mm, Sartorius Biolab Products, France) and incubated on DMB agar plates at 28 °C for 4 h to obtain a monolayer of bacteria. Then the membranes covered by bacteria were laid on agar one-well dishes (size 127.8 × 85.5 mm, Nunc, Thermo Scientific, Montigny le Bretonne, France), which were directly transferred into the gas delivery device [28].

### 2.2. Disruption of the hmp gene in P. fluorescens MFAF76a

MFAF76aΔ*hmp* was generated by an in frame central deletion (711 bp) in *hmp* (1182 bp; KR818822). This mutation was achieved by PCR using the *Muta1-hmp* and *Muta2-NdeI-hmp* (781 bp product) primers and *Muta3-NdeI-hmp* and *Muta4-XbaI-hmp* primers (793 bp product) (Table 2).

Hybridization temperature (Tm) was evaluated for each primer using NEB Calculator [29]. Each PCR was performed with the Phusion^®^ High-Fidelity DNA polymerase (M0530S, NEB, Evry, France) as recommended by the provider. The obtained PCR products corresponding to the upstream and downstream parts of the *hmp* were both digested by *Nde*I (R0111S, NEB) and ligated by T4 DNA ligase. Then, a third PCR was conducted using *Muta1-hmp* and *Muta4-XbaI-hmp* primers to amplify Δ*hmp*. The product obtained was digested by *Xba*I, then inserted into pAKE604 [25] that had been linearized by digestion with *Sma*I (R0141S, NEB, Evry, France) and *Xba*I (R0145S, NEB, Evry, France) to produce the plasmid pAKE604Δ*hmp*. The plasmid was then sequenced and transferred into MFAF76a through the biparental mating method. MFAF76a cells and *E. coli* S17.1 [24] containing pAKE604Δ*hmp* were mixed before being spotted onto LB agar plates and incubated at 37 °C overnight. The resulting mating mixture was re-suspended in 1 mL of sterile saline solution and 0.1 mL aliquots were spread on cetrimide agar plates (Difco™ BD 285420, Fisher scientific, Illkirch, France) supplemented with 50 µg/mL kanamycin (11815024, ThermoFisher, Leiden, Netherlands), to select *P.s* carrying the integrated modified plasmid. Then the second recombination event was obtained by plating bacteria on LB agar medium supplemented with 10% sucrose (S25590, Fisher scientific). The mutant containing the in frame central deletion in the *hmp* gene was verified by DNA sequencing and named MFAF76a-Δ*hmp*.

### 2.3. hmp-Complementation of MFAF76a-Δhmp

The *hmp* gene was amplified by PCR using the *hmp-EcoRI-F*/*hmp-XbaI-R* primers (Table 2) with the Phusion^®^ High-Fidelity DNA Polymerase under standard conditions. NEB Tm calculator was used to calculate the primer hybridization temperatures. The pPSV35 shuttle vector [26] and the amplified *hmp* gene were digested by *Eco*RI (R0101S, NEB) and *Xba*I, generating cohesive ends. T4-DNA-ligase (M0202S, NEB) was used to insert the amplified fragment into pPSV35 downstream of the PlacUV5 promoter. *E. coli* Top10^®^ were used transformed by thermal shock with the plasmid pPSV35-*hmp.* The plasmid DNA was extracted by the GeneJET Plasmid Miniprep Kit (K0702, ThermoFisher Scientific), amplified by PCR using plasmid-specific primers and the resulting amplicon was verified by sequencing. Fresh colonies of MFAF76a or MFAF76a Δ*hmp* mutants were washed twice with 1 mL of cold sterile water before being resuspended in 100 μL of cold sterile water. 100 ng of plasmid (pPSV35 or derivatives) were added to the solution, and the cells were elecroporated in 1-mm electroporation cells at 1.8 kV for 5 ms (GTF100 Gene Transformer, Savant Inc., Holbrook, AZ, USA). 900 µL of LB were added to the solution, and the mixture was incubated at 28 °C for 1 h with shaking at 180 rpm. Transformed bacteria were then plated on LB-agar supplemented with gentamycin for selection.

### 2.4. Exposition to NO_2_ and NO

As previously described by Kondakova et al., an exposure system was developed in order to mimic the environmental exposition [30]. Briefly, the bacterial beds were exposed simultaneously during 2 h at 28 °C to a constant gas stream (2 L/min) in two separate exposure chambers. The first chamber, corresponding to the control was swept by synthetic air. In the second chamber, NO_x_-exposed bacteria were laid in contact with a mixture of N_2_/O_2_ 8/2 (*v*/*v*) complemented with 45 ppm of NO_2_ (Air Liquide GMP Europe) or 45 ppm of NO (Air Liquide GMP Europe). After exposure, all bacteria were resuspended in sterile saline solution.

### 2.5. Cultivability Assays

Cultivability tests were performed by a serial dilution in saline solution from the bacterial suspension to reach a dilution of 10^−7^. A total of 100 μL of dilution range 10^−4^ to 10^−7^ were spotted onto LB agar plates in triplicate for both conditions. After incubation at 28 °C for 24 h, viable colony forming unit were numerated.

### 2.6. Flow Cytometry Assay

After exposure, 1 mL of bacterial suspension was stained for 15 min with SYTO9 (5.01 nM) and propidium iodide (PI, 30 nM) using the Live/Dead BacLight kit (L-7012, ThermoFisher). Thence, damaged and total bacteria were respectively observed as PI and SYTO9 positive using a flow cytometer (CytoFLEX S, Beckman Coulter, Roissy, France) and the CytExpert v1.2 software. PI and SYTO9 were excited at the fixed wavelength of 488 nm and their fluorescence were detected at 690 ± 50 nm and 525 ± 40 nm, respectively. For each measurement, 10,000 events were collected at a medium flow (30 µL/min). A negative control for membrane permeability was performed on bacterial culture without exposure. Positive control was realized using cells treated with ethanol 50% (10375842, Fisher scientific) for 10 min before PI staining and calibration of the PI threshold on the software.

### 2.7. RNS Labelling

After exposure, bacterial suspension was centrifuged for 5 min at 13,000× *g* and cells were washed twice with physiological water and then resuspend at 580 nm OD of 1. The cell and supernatant fractions were then labelled separately with fluorescent probes specific to peroxynitrite (ONOO^−^) and nitric oxide (NO) using 1,2,3-dihydrorhodamine (DHR, D1054, Sigma, Saint-Quentin-Fallavier, France) at 7.2 mM and diaminofluorescein-FM diacetate (DAF-FM DA, D1946, Sigma, Saint-Quentin-Fallavier, France) at 5 mM respectively. The kinetic of ONOO^−^ and NO production was monitored each 15 min at excitation/emission wavelength of 500/540 nm and of 490/530 nm, respectively using a microplate reader (Spark^®^ Cyto, TECAN™) at 28 °C with shaking at 180 rpm for 6 h. Nitrite (NO_2_^−^) concentration was determined through the Griess reaction by adding an A solution (5 N acetic acid—695092, Sigma—+ 0.8% sulfanilic acid—251917, Sigma) and a B solution (5 N acetic acid + 0.6% Alpha-Naphthylamin—N9005, Sigma) to the suspension. Data were then normalized to the wild-type (WT) control condition. The production of NO_2_^−^ was monitored by absorbance at OD 520 nm. An internal control was conducted without labelling cells or supernatant fraction.

### 2.8. Quantitative Reverse-Transcription Real Time Polymerase Chain Reaction (RT-qPCR)

Total RNAs were extracted from pellets of bacteria either exposed to 45 ppm NO_2_ or synthetic air (control) using the RNAprotect^®^ Bacterial reagent and RNeasy^®^ Mini kits (QIAGEN, Hilden, Germany). The Turbo DNA-free kit (Invitrogen, Vilnius, Lithuania) was used to eliminate residual genomic DNA. Isolated RNAs were then converted to single stranded cDNAs in a non-specific manner using the High-Capacity cDNA Reverse Transcription Kit (Applied Biosystems, Brumath, France) using manufacturer’s instructions. The RT-qPCR experiments were realized as described in Gicquel et al. [31] using the primers listed in Table S1. Analysis of the relative quantification of the mRNAs between the two conditions was performed by the comparative CT (2^−ΔΔCT^) method as previously described in Guyard-Nicodème et al. [32]. The internal standard used in these experiments was *recA* gene expression, which is stable within the samples to be compared regardless of the experimental conditions [33].

### 2.9. Whole Proteome Identification and Quantification

Bacterial suspensions were centrifuged at 13,000× *g*, 4 °C during 20 min after NO_2_ exposure. Then, bacterial pellets were re-suspended in a 20 mM Tris-HCl buffer (pH 7.4) and sonicated. Bacterial lysates were centrifuged (10,000× *g*; 10 min; 4 °C). Protein concentration was measured using a Bradford test (Biorad™, Marnes-la-Coquette, France). The separation, identification and relative abundance of each protein was carried out as previously described [34]. Briefly protein extracts were concentrated on an SDS-PAGE (7%), stained using Coomassie dye, before dehydration of protein bands and trypsin digestion. Finally, the identification was achieved using a LTQ Orbitrap Elite (Thermo Scientific) coupled to an Easy nLC II system (Thermo Scientific). The mobile phase consisted of H_2_O/0.1% formic acid (FA) (buffer A) and ACN/FA 0.1% (buffer B). Samples were injected onto an enrichment column (C_18_ PepMap100, Thermo Scientific) and eluted at 300 nL/min with a three-step linear gradient (from 2 to 40% B over 75 min, from 40 to 80% B in 4 min and 11 min at 80% B). The mass spectrometer was operated in positive ionization mode and the capillary voltage and source temperature were respectively set at 1.5 kV and 275 °C. Sample analysis was performed using CID (collision induced dissociation) method. Fragmentation happened in the linear ion trap analyzer with a collision energy of 35%. Orbitrap analyzer measurements were performed using on-the-fly internal recalibration (lock mass) at m/z 445.12002 (polydimethylcyclosiloxane). Raw data after MS analysis were processed using the Progenesis LC-MS software (Nonlinear Dynamics). The retention times of all samples were aligned on one reference sample within the experiment. One-way analysis of variance (ANOVA) calculations was performed on aligned and normalized data. Peptide features with a *p*-value and a *q*-value inferior to 0.05, and with a power superior to 0.8 were retained. The MS/MS spectra from retained peptides were identified using Mascot (Matrix Science), against the database restricted to *P. fluorescens* A506 [35]. Proteins were retained if their fold-change varied by 1.8-fold or more between the two experimental conditions.

### 2.10. Statistical Analyses

All experiments were repeated at least three times. Two tailed unpaired *t*-test was used to determine the significances of differences between mean values for the cultivability, live/dead cytometry and RT-qPCR experiments. Significance was set at *p* < 0.05 (*), *p* < 0.01 (**) and *p* < 0.001 (***).

## 3. Results

### 3.1. NO_2_ Exposure Leads to a Global Response to ROS and RNS While NO Exposure Leads to an Hmp-Focused Response

**Figure 1:**
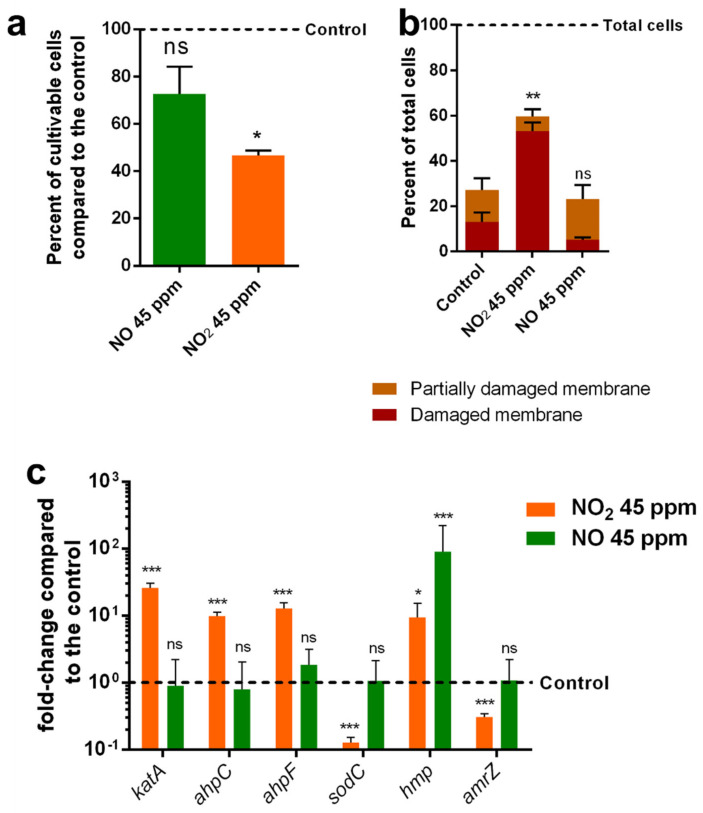
Physiological modifications of *P. fluorescens* MFAF76a after exposure to NO_2_ or NO. (**a**) Cultivability of bacteria after exposure to NO_2_ (in orange) or NO (in green) compared to cells exposed to synthetic air. (**b**) Membrane integrity assessed by live-dead flow cytometry assays using Live/Dead BacLight kit (L-7012, ThermoFisher) according to the manufacturer’s recommendations. (**c**) Expression of genes encoding proteins involved in RNS and ROS detoxification. Graphs represent means ± SEM. ns = *p* > 0.05; * = *p* < 0.05; ** = *p* < 0.01; *** = *p* < 0.001, N ≥ 3.

Previous work showed significant alterations of MFAF76a morphology after exposure to 45 ppm of NO_2_ [22]. Here, we compared the differences in the effects of NO_2_ and NO at this concentration on this bacterial strain. Similarly to what was previously described [36], exposure to NO_2_ led to a 53.3% reduction of cell surviving (Figure 1a). However, exposure to NO did not lead to a significant reduction in bacterial cultivability. To further characterize the physiological effects of these molecules on *P. fluorescens*, membrane integrity assays were performed by flow cytometry using the Live/Dead BacLight kit (L-7012, ThermoFisher) composed of SYTO9™, a membrane-permeant DNA probe with green emission fluorescence, and Propidium iodide (PI), a membrane impermeant DNA probe emitting red fluorescence (Figure 1b). After labelling, three populations were distinguishable. A first population of cells was labelled by SYTO9™ only, characteristic of cells presenting an intact membrane. A second population was labelled with PI and only slightly labelled by SYTO9™, similarly to the control permeabilized cells exposed to 50% ethanol, characteristic of cells presenting damaged membranes (noted damaged membranes). A third more heterogeneous population was strongly labelled by both probes. This third profile was not found in the permeabilized cells control, suggesting that these cells were only partially permeabilized. Results showed that exposure to NO_2_ led to a strong increase in the number of cells with damaged membranes, from 13.1% to 53.1%, while no significant difference was observed after exposure to NO (Figure 1b). The proportions of partially damaged cells did not significantly change after exposure to the two gases. As a result, the overall number of cells with altered membranes was significantly increased in NO_2_ exposed bacteria (Figure 1b). To assess the cellular response to these molecules, a RT-qPCR analysis of the transcription of several genes involved in the response to nitrosative and oxidative stresses was performed (Figure 1c). This analysis focused on five genes involved in the chelation or detoxification of ROS and RNS *katA*, *ahpCF*, *sodC* and *hmp*, which encode the catalase KatA, the alkyl-hydroperoxidase AhpCF, the superoxide dismutase SodC and the flavohemoprotein Hmp, respectively. *amrZ* codes for the global transcriptional regulator AmrZ involved in environmental adaptation and regulation of various virulence factor genes [37,38]. NO_2_ exposure led to a significant increase in the transcription of *katA*, *ahpC*, *ahpF* and *hmp* and a significant underexpression of *sodC* and *amrZ*. NO exposure, despite its apparent absence of physiological effect, still induced a strong induction of the transcription of *hmp* (Figure 1c).

### 3.2. Hmp Is Involved in the Preservation of Membrane Integrity after Exposure to NO_2_

**Figure 2:**
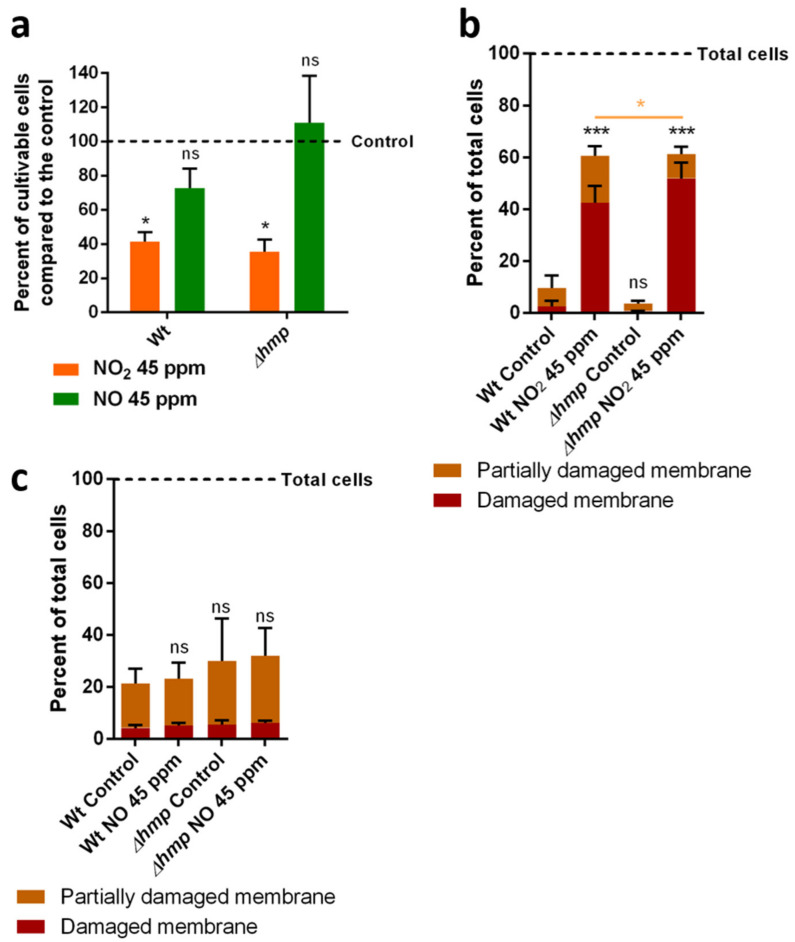
Impact of *hmp* deletion on the physiological modifications of *P. fluorescens* MFAF76a after exposure to NO_2_ or NO. (**a**) Cultivability of bacteria after exposure to NO_2_ or NO compared to cells exposed to synthetic air. (**b**) Membrane integrity of *P. fluorescens* MFAF76a and MFAF76a Δ*hmp* assessed by the Live/Dead BacLight kit (L-7012, ThermoFisher) flow cytometry assays after exposure to 45 ppm of NO_2_. (**c**) Membrane integrity of *P. fluorescens* MFAF76a and MFAF76a Δ*hmp* assessed by live-dead flow cytometry assays after exposure to 45 ppm of NO. Graphs represent means ± SEM. ns = *p* > 0.05; * = *p* < 0.05; *** = *p* < 0.001, N ≥ 3. In cytometry experiments, black stars represent the significance of overall cell membrane alterations, while orange stars represent the difference between partially damaged membranes between the wild-type and the Δ*hmp* mutant.

Considering the transcription increase in *hmp* in both the NO_2_ and NO conditions, its impact on the bacterial physiology in response to these RNS was studied using a deletion mutant (Δ*hmp*). The growth rate of the wild-type and the Δ*hmp* mutant are identical (data not shown). While the cultivability of both the wild-type (WT) and deletion mutant decreased after exposure to NO_2_, no significant difference was found between the two (Figure 2a). However, the amount of partially damaged cells in response to NO_2_ was decreased in favor of the damaged cells (Figure 2b). No difference was found between the control cells and the cells exposed to NO (Figure 2c)

### 3.3. NO_2_ Exposure Induces the Formation of Derived Nitrogen Species Unlike NO

**Figure 3:**
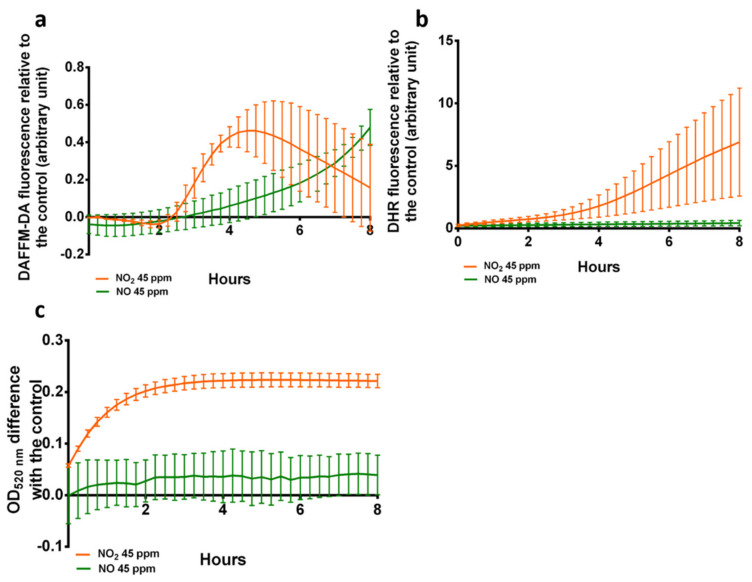
Observation of NOx formation after NO_2_ and NO exposure in the bacterial supernatant. (**a**) Kinetics of DAFFM-DA fluorescence in response to NO after exposure to NO and NO_2_. (**b**) Kinetics of DHR fluorescence in response to ONOO^−^ after exposure to NO and NO_2_. (**c**) Optical density at 520 nm of Griess reagent in response to NO_2_^−^. N = 3.

To understand the differences observed between the cells exposed to NO_2_ and NO, the formation of the derived RNS species in the cell supernatant after exposure to NO_2_ was assessed (Figure 3) as no signal could be detected within washed cells. The formation of NO, ONOO^−^ and NO_2_^−^ after exposure to NO or NO_2_ was monitored using the fluorescent probes DAFFM-DA (Figure 3a) and DHR (Figure 3b), and the colorimetric Griess reagent (Figure 3c), respectively. NO was detected after exposure to both NO and NO_2_, but ONOO^−^ and NO_2_^−^ were only detected after exposure to NO_2_.

### 3.4. The Bacterial Response to NO_2_ Is Dependent of Its Concentration

**Figure 4:**
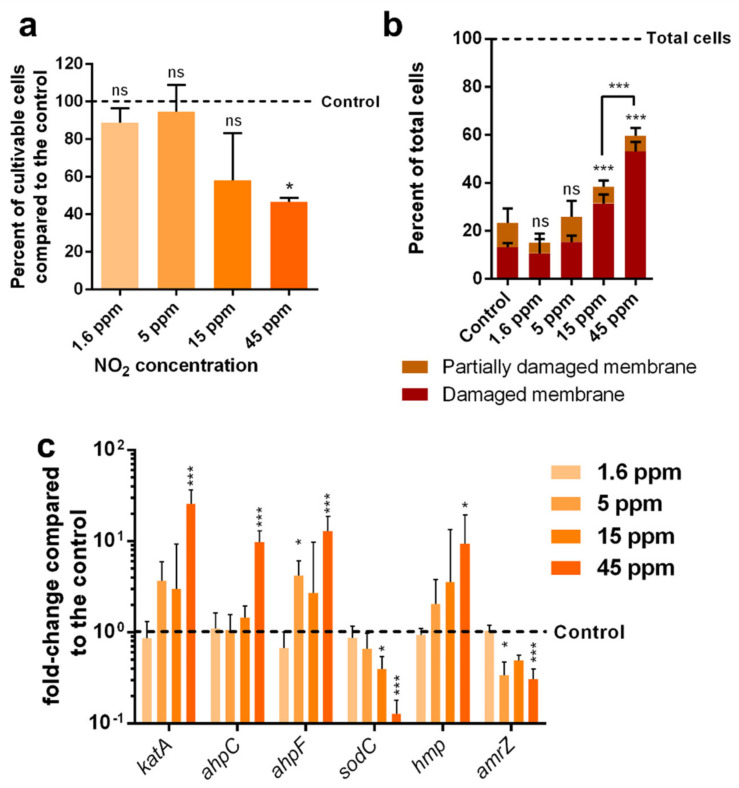
Physiological modifications of *P. fluorescens* MFAF76a after exposure to various concentrations of NO_2_. (**a**) Cultivability of bacteria after exposure to NO_2_ compared to cells exposed to synthetic air. (**b**) Membrane integrity assessed by live-dead flow cytometry assays using Live/Dead BacLight kit (L-7012, ThermoFisher) according to the manufacturer’s recommendations. (**c**) Expression of genes coding for proteins involved in RNS and ROS detoxification. Graphs represent means ± SEM. ns = *p* > 0.05; * = *p* < 0.05; *** = *p* < 0.001, N ≥ 3.

To determine the concentration of NO_2_ inducing a cellular response, bacteria were exposed to NO_2_ concentrations of 1.6, 5, 15 and 45 ppm. For each of the four concentrations, the cell viability, membrane integrity and transcriptional response were assessed using similar protocols to the cells exposed to NO and NO_2_ at 45 ppm (Figure 4). Cell viability was significantly decreased at 45 ppm (Figure 4a), and membrane integrity was significantly impaired at 15 ppm, and further significantly impaired at 45 ppm (Figure 4b). The transcriptional modifications of genes involved in ROS and RNS detoxifications were significant at 5, 15 or 45 ppm (Figure 4c).

### 3.5. Exposure to NO_2_ Leads to Production Alterations of Proteins Involved in RNS- and ROS-Mediated Damages

**Figure 5:**
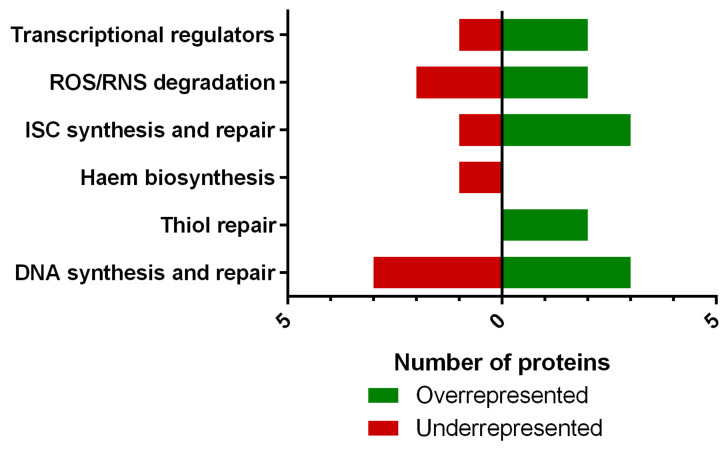
Proteomic profile of *P. fluorescens* MFAF76a after NO_2_ exposure. ROS/RNS: reactive oxygen/nitrogen species; ISC: iron-sulfur cluster. The identification of proteins was performed using *P. fluorescens* A506 as reference (N = 4).

To better understand MFAF76a response to NO_2_ exposure, a proteomic analysis of the strain after exposure to 45 ppm of NO_2_ was performed (Figure 5 and Table 3). Results notably showed the modulation of proteins involved in (i) citrate cycle, (ii) amino acid synthesis and degradation, (iii) DNA synthesis and repair and (iv) protein structuration such as iron sulfur cluster and haem biosynthesis or thiol repair, which are targets of RNS. Over-represented proteins and under-represented proteins were respectively 2 and 5 for the citrate cycle, 8 and 19 for amino acid metabolism, 3 and 3 for DNA synthesis, 2 and none for DNA repair, 4 and 1 for clusters, none and 2 for haem biosynthesis and 3 and 1 for thiol biosynthesis.

## 4. Discussion

Nitrosative stress is regularly encountered by bacteria, notably in the context of the immune response, and must be countered by bacteria. The two main RNS that airborne bacteria are exposed to are NO_2_ and, usually in lesser quantity, NO [22]. To study the specific impact of these two species on bacteria, the airborne strain MFAF76a was exposed to 45 ppm of NO_2_ or 45 ppm of NO for 2 h via an exposition system described previously [30]. A control was realized with synthetic air.

First, the cultivability of bacteria under these two pollutants was assessed, showing that NO_2_, as previously described [36], induces cell mortality, while no effect was found for NO alone. This suggests that NO_2_ and NO have drastically different effects on the cells. These results were coherent with the fact that NO did not alter the cell membrane integrity, while NO_2_ induced a large increase in damaged cell membranes. To understand the cellular response to these alterations, the transcription of several genes coding for proteins involved in the detoxification of RNS and ROS and virulence were analyzed. Transcription of the *amrZ* gene, coding for the transcriptional regulator AmrZ, was repressed after exposure to NO_2_. AmrZ is involved in the regulation of cell mobility and several virulence factors such as extracellular polysaccharide production, bis-(3′,5′)-cyclic diguanylate (c-di-GMP) and flagella [37,39,40,41]. Exposure to NO_2_ leads to modifications in the expression of a wide array of these genes, despite not being known to induce them. Expression of *katA*, *ahpC* and *ahpF* is notably known to be under the transcriptional control of OxyR, which can sense nitrosative stress alterations such as S-nitrosothiols [42]. Likewise, *hmp* is over-expressed after exposure to NO_2_, although its transcriptional regulators are known to be sensitive to NO [43,44,45]. Interestingly, despite an absence of apparent physiological effect, exposure to NO strongly and specifically induced the transcription of *hmp* in MFAF76a. The overexpression of *hmp* after exposure to both NO and NO_2_ suggests that Hmp, known to detoxify NO, plays an important role in the cell resistance to these two stresses. Both the *hmp* deletion mutant Δ*hmp* and the wild strain suffered a similar loss of cultivability after exposure to 45 ppm of NO_2_, while no loss of cultivability was found after exposure to NO. However, the number of cells with partially damaged membranes after exposure to NO_2_ was significantly reduced in Δ*hmp* compared to the wild-type in favor of completely permeabilized cells. This indicates that Hmp plays a minor role in membrane protection against NO_2_. Deletion of *hmp* did not affect the bacterial response to NO, suggesting that 45 ppm of NO are not sufficient to induce significative alterations of cell physiology even in the Δ*hmp* mutant lacking part of its resistance mechanisms.

Since RNS are very reactive species, they often form a network of derivative species. Differences between the derivative species produced by NO_2_ and NO could potentially explain the differences observed in the effects and transcriptional profiles between the two conditions. To explore this hypothesis, three possible products of NO_2_ and NO reactions were measured after exposure. NO presence was assessed using DAFFM-DA, ONOO^−^ was assessed through DHR, and NO_2_^−^ was assessed through the Griess reagent. Since the fluorescence of the probes depends on their reaction with RNS, the kinetics reflected in the curves do not necessarily represent the kinetics of the RNS formation, but they can inform on the presence or absence of such species in the cell. Results showed that exposure to NO_2_ leads to the apparition of the three species in the supernatant. While ONOO^−^ is not a direct product of NO_2_ reactions, it could be formed indirectly through reactions with other cell components. Indeed, cells exposed to NO_2_ presented a yellow color typical of nitrated tyrosines [6]. After exposure to NO, NO was detected in the medium through DAFFM-DA fluorescence but did not lead to the production of either ONOO^−^ or NO_2_^−^. No RNS were detected in the cell fraction (data not shown), probably due to RNS leakage out of the cells during the centrifugation and/or resuspension processes, as these species are able to cross membranes directly or through membrane transporters. This indicates that NO_2_ exposure leads to the formation of the highly reactive ONOO, known for its cytotoxicity, which in turn can generate other RNS and ROS. This could explain the physiological effects of NO_2_ and the global response observed by RT-qPCR. Similarly, NO exposure does not seem to generate other reactive species, explaining its lack of significant effect and the specificity of the transcriptional response observed. It can be noted that the mass transfer specificities of each of these molecules into biofilms could lead to a different response in bacteria in biofilm form.

As with all chemical stresses, the concentration of NO_2_ is a crucial parameter in the toxicity of the molecule. To determine the concentrations of NO_2_ able to generate acute nitrosative stress in MFAF76a and to assess whether the cell response is dose-dependent, cultivability, membrane integrity and RT-qPCR assays were performed on cells exposed to concentrations of NO_2_ ranging from 1.6 to 45 ppm. Cell cultivability was only significantly reduced at 45 ppm, although a tendency to decline was observed from 15 ppm. These results are coherent with the membrane integrity assay, which showed an increase in the proportion of cells with damaged membranes at 15 ppm, which was more important at 45 ppm. Lower concentrations did not significantly affect either cultivability or membrane integrity, although we cannot exclude that chronic exposure to RNS could impact the cells in such a way. RT-qPCR assay showed that the cellular response to NO_2_ is different in function of its concentration. At 5 ppm, *amrZ* is under-expressed at a level staying constant with increasing concentrations. *ahpF* is overexpressed at 5 ppm, suggesting AhpCF is prioritized over the KatA catalase for detoxifying H_2_O_2_ at low concentrations, although *ahpC* is intriguingly only over-expressed at 45 ppm of NO_2_. Intriguingly, *sodC* is under-expressed from 15 ppm of NO_2_, which might be related to its reliance on metallic ions (Cu^2+^ and Zn^2+^) as discussed below. Finally, *katA* and *hmp* were only significantly over-expressed at 45 ppm of NO_2_.

After exposure to 45 ppm of NO_2_, various proteins involved in the detoxification of ROS and RNS and the synthesis and repair of their biological targets were modified.

Firstly, two major nitrosative and oxidative stress regulators were modified after exposure to NO_2_. Anr, a major sensor of oxygen availability and sensor of oxygen and nitrogen presence [46] was over-represented by 3.4-fold after exposure to NO_2_, while the iron metabolism repressor Fur was under-represented by 4.6-fold, suggesting an important implication of the pathways regulated by these sensors in response to NO_2_. Accordingly, the iron sulfur cluster (ISC) biosynthesis and repair pathway was heavily modified after exposure to NO_2_. The methionine biosynthesis regulator MetR, involved in resistance to nitrosative stress through the regulation of Hmp [47] was also over-represented by 1.8-fold.

Three proteins involved in the degradation of oxidative and nitrosative stress mediators were modulated after exposure to NO_2_. The catalase KatE was under-represented by a 7.8-fold, while a peroxyredoxin of the AhpC family was over-represented by 2.1-fold and the thiol peroxidase Tpx was over-represented by 3.7-fold. While these three proteins both convert the dangerous hydrogen peroxide into water and oxygen, KatE possesses an ISC, while AhpC and Tpx do not. The shift between KatE and Tpx/AhpC family peroxyredoxin could therefore be a way to avoid the use of ISCs in this process, to both limit the number of ROS-inducing Fenton reactions and redirect the use of functional ISCs to other processes. In addition, NirB, a subunit of the nitrate reductase NirBD containing an ISC was also under-represented after exposure to NO_2_ by a 3.3-fold.

After exposure to NO_2_, the abundances of IscR, IscU and HscA are increased, suggesting an increase in ISC biosynthesis and repair. In *E. coli*, IscR usually represses the transcription of *isc* operon under physiological conditions. However, IscR contains a 4Fe-4S cluster sensitive to RNS and ROS which is essential for its repressor activity. Thus, an exposure to NO_2_ could promote an increase in ISC biosynthesis. However, IscS is significantly under-represented. IscS possesses a cysteine residue (Cys^238^) on its active site critical for its sulfur-transfer activity [48]. Thus, similarly to AhpC, IscS could endorse a post-translational modification through S-nitrosylation leading to the degradation of the misfolded protein. Decrease in IscS protein quantity implies a diminution in transfer of sulfur to the nascent ISC which impairs further step formation of ISC. Furthermore, heme biosynthesis could be impaired after NO_2_ exposure. Indeed, HemX (PA5258) and HemY (PA5257) proteins responsible for the formation of protoporphyrin IX ring are decreased. In *P. aeruginosa*, PrrF1/2 induces the expression of a non-coding PrrH mRNA, known to regulate heme homeostasis [49].

Furthermore, several thiol-containing proteins are sensitive to ROS/RNS stress. Thus, the thiol-reparation appears to be an essential process in oxidative and nitrosative stress tolerance. In this study, a significant over-production of thioredoxin (TrxA) is observed. TrxA is able to repair nitrosylated thiols in proteins [50]. In *P. aeruginosa*, the regulation of *tpx* and *trxA* expression is ROS-dependent, but is mediated differentially by OxyR [51] and IscR [52], respectively. GrxA, a glutaredoxin OxyR-dependent, is also over-produced after NO_2_ exposure [51]. GrxA, coupled with the glutathione-S transferase, repairs protein containing an altered thiol-residue following oxidative stress [53].

Furthermore, the proteins catalyzing the first step of the purine and pyrimidine synthesis pathway through salvage, xanthine phosphoribosyltransferase and uracil phosphoribosyltransferase respectively, are both increased, while the proteins involved in the first steps of nucleotide de novo biosynthesis (deoxycytidine triphosphate deaminase, amidophosphoribosyltransferase and bifunctional purine biosynthesis protein PurH) are decreased, with the exception of the phosphoribosylaminoimidazole carboxylase ATPase subunit, which is over-represented. This switch toward the salvage pathway favors the repair of nucleotides potentially impacted by the ROS and RNS derived from NO_2_. In addition, recombinase RecA and the DNA gyrase subunit A, involved in DNA repair, are increased after NO_2_ exposure. Collectively, these proteomic results suggest the activation of repair pathways of most major biomolecules in response to NO_2_, with important changes in ISC synthesis and use in the cell. In addition to these results, several alterations regarding the envelope components of MFAF76a were noted and discussed somewhere else [22].

Overall, these results show that NO_2_ and NO, despite existing in an equilibrium in the atmosphere, can have drastically different effects on airborne bacteria. Exposure to concentrations of NO_2_ of 5 ppm elicit a bacterial response to oxidative and nitrosative stress, with significant physiological alterations at 15 ppm and over. NO exposure does not have noticeable physiological effects on MFAF76a, although it leads to a specific response through the protein, Hmp. The difference between the effects of these two molecules on MFAF76a can be partially explained by the production of derivate RNS by NO_2_, which are absent after exposure to NO. Finally, the proteomic analysis showed that MFAF76a, in addition to the production of detoxification enzymes, responds to elevated nitrosative stress through the activation of repair pathways of both proteins and DNA, and a shift of iron-sulfur cluster management.

## Figures and Tables

**Table 1:** Strains and plasmid used in this study. Gm^R^: gentamycin resistance; Km^R^: kanamycin resistance, Ap^R^: Ampicillin resistance.

Strains/Plasmids	Relevant Phenotype/Genotype	Reference
***P. fluorescens***		
MFAF76a (WT)	Airborne isolate, able to grow at 37 °C	[23]
MFAF76aΔ*hmp* (Δ*hmp*)	MFAF76a with a central deletion of in *hmp* gene (711 bp)	This study
MFAF76aΔ*hmp* + pPSV35 (EV)	MFAF76aΔ*hmp* with pPSV35 empty vector, Gm^R^	This study
MFAF76aΔ*hmp* + *hmp* (*hmp^+^*)	MFAF76aΔ*hmp* with pPSV35 carrying wild-type *hmp* gene, Gm^R^	This study
***E. coli***		
S17.1	RP4-2-Tc::Mu, *aph*::Tn7, *recA*, Sm^R^, donor strain for conjugation	[24]
Top10^®^	F-*mcrA*, Δ(*mrr*-*hsd*RMS-*mcr*BC), Φ80*lacZ*ΔM15, Δ*lac*X74, *recA*1, *araD*139, Δ(*araleu*)7697, *galU*, *galK*, *rpsL*, (StrR), *endA1*, *nupG*	ThermoFischer Scientific
***Vectors***		
pAKE604	Conjugative suicide vector; Km^R^, Ap^R^, *oriT*, *lacZ*, *sac*B	[25]
pPSV35	*Pseudomonas aeruginosa oriV*, *lacI*^q^ *mob*+, P*lac*UV5, pUC18MCS, expression vector, Gm^R^	[26]

**Table 2:** List of primers used for the mutation of *hmp* in MFAF76a. In bold cases are represented the restriction site for *Nde*I, *Xba*I and *Eco*RI in primers *Muta2-NdeI-hmp*, *Muta3-NdeI-hmp*, *Muta4-XbaI-hmp*, *hmp-EcoRI-F* and *hmp-XbaI-R* respectively, Tm: hybridization temperature in Celsius degree.

Primer Name	Primer Sequence (5′→3′)	Tm (°C)
***Mutagenesis primers***		
*Muta1-hmp*	CATCGACGAATAAAGGACAG	59
*Muta2-NdeI-hmp*	TAATAA**CATATG**ATGATTTTGGCCACCA	57
*Muta3-NdeI-hmp*	TAATAA**CATATG**CTATTGCTATGCCGAAGAAG	58
*Muta4-XbaI-hmp*	TAATAA**TCTAGA**CGGCAGATCATCGACAAT	61
***Surexpression primers***		
*hmp-EcoRI-F*	TAATAA**GAATTC**AGTCACCTTATGCTTAGCG	56
*hmp-XbaI-R*	TAATAA**TCTAGA**TTGCCTTTCCTTTGTAAGTC	57

**Table 3:** Proteins involved in sensing, degrading and repairing RNS and ROS alterations after exposure to 45 ppm of NO_2_. * = *p* < 0.05; ** = *p* < 0.01, *** = *p* < 0.001, N = 3.

	Accession	Protein	Fold Change	Anova (*p*)	
**Transcriptional regulators**	PflA506_3874	transcriptional regulator Anr	3.4	5.4 × 10^−3^	**
	PflA506_4567	ferric uptake regulation protein Fur	−4.6	5.8 × 10^−3^	**
	PflA506_1087	transcriptional regulator MetR	1.8	4.6 × 10^−3^	**
**ROS/RNS degradation**	PflA506_0070	catalase HPII KatE	−7.8	1.0 × 10^−2^	*
	PflA506_1119	AhpC/TSA family antioxidant protein	2.1	1.2 × 10^−3^	**
	PflA506_3912	nitrite reductase NirB large subunit	−3.3	9.9 × 10^−5^	***
	PflA506_2948	thiol peroxidase Tpx	3.7	8.9 × 10^−6^	****
**Iron Sulfur cluster**	PflA506_4376	iron-sulfur cluster assembly transcription factor IscR	2.4	7.6 × 10^−4^	***
	PflA506_4375	cysteine desulfurase IscS	−2.6	2.1 × 10^−3^	**
	PflA506_4374	FeS cluster assembly scaffold IscU	2.4	4.0 × 10^−3^	**
	PflA506_4371	Fe-S protein assembly chaperone HscA	2.6	2.1 × 10^−3^	**
**Haem Biosynthesis**	PflA506_5216	HemY protein	−2.1	5.1 × 10^−3^	**
**Thiol repair**	PflA506_1116	monothiol glutaredoxin Grx	7.2	1.4 × 10^−2^	*
	PflA506_5190	thioredoxin TrxA	8.0	5.5 × 10^−3^	**
**DNA synthesis and repair**	PflA506_5246	xanthine phosphoribosyltransferase	2.3	2.9 × 10^−4^	***
	PflA506_0886	uracil phosphoribosyltransferase	2.2	7.6 × 10^−5^	***
	PflA506_1266	deoxycytidine triphosphate deaminase	−2.2	4.1 × 10^−3^	**
	PflA506_3496	amidophosphoribosyltransferase	−2.2	9.8 × 10^−3^	**
	PflA506_0592	bifunctional purine biosynthesis protein PurH	−2.8	5.3 × 10^−3^	**
	PflA506_5335	phosphoribosylaminoimidazole carboxylase ATPase subunit	2.0	1.1 × 10^−3^	**

## Data Availability

Data is contained within the article or Supplementary Materials.

## Supplementary Materials

The following supporting information can be downloaded at: https://www.mdpi.com/article/10.3390/microorganisms10081576/s1, Table S1: List of primers used in the RTqPCR experiments.
