# Supplementary material for: Detoxification Response of Pseudomonas fluorescens MFAF76a to Gaseous Pollutants NO2 and NO
Source: Microorganisms. 2022 Aug 5;10(8):1576. doi: 10.3390/microorganisms10081576 (PMC9414441; doi:10.3390/microorganisms10081576)
Supplement: Supplementary file 1 [file microorganisms-10-01576-s001.zip › microorganisms-1820718-Supplementary.pdf]

Table S1. List of primers used in the RTqPCR experiments.

Gene	Forward Primer	Reverse Primer
ahpC	GCCTATCATCAACAGCCAAGTAAA	AGCATCCGAGACTTGAACGAA
ahpF	CGAACACCGATTGGCTGAA	ATCCCCGCAATCGAAGTCT
amrZ	AGGTGGCTCGCAATCATCAT	GTGCGCCTTCCTGAATAAGACT
hmp	GCGTCCAGCTATCTGCATGA	ACGGCGGGAACAGTTGAA
katA	CAAGCGGCGGCTCATTA	CGATCAACGGAAACTCCTTCTG
sodC	GCAACGGAGAAAGTCGCAAT	ATATGGCGTCTCACTGACTGTTACC
